# The effect of fasting on spirometry indices and respiratory symptoms in asthmatic patients

**DOI:** 10.34172/jcvtr.2022.21

**Published:** 2022-06-28

**Authors:** Mohammad Reza Ghaffary, Ali Talei, Maryam Moradian, Shamsi Ghaffari

**Affiliations:** ^1^Tuberculosis and Lung Disease Research Center, Tabriz University of Medical Sciences, Tabriz, Iran; ^2^Department of Internal Medicine, School of Medicine, Tabriz University of Medical Sciences, Tabriz, Iran; ^3^Rajaie Cardiovascular Medical and Research Center, Iran University of Medical Sciences, Tehran, Iran; ^4^Cardiovascular Research Center, Tabriz University of Medical Sciences, Tabriz, Iran

**Keywords:** Asthma, Fasting, Ramadan, Spirometry

## Abstract

***Introduction:*** Ramadan can alter the course of diseases by changing nutrition patterns, sleep habits, and medication-taking schedules. There are some concerns that patients with asthma may be affected by these alterations during Ramadan and experience deterioration of their symptoms. This study aimed to investigate the effect of fasting in Ramadan on the severity of the disease and spirometric parameters in patients with asthma.

***Methods:*** An overall 120 patients with moderate to severe asthma were investigated during Ramadan and categorized into two groups of fasting (60 cases) and non-fasting (60 cases) groups. Patients underwent spirometry before and after Ramadan and asthma control status was also assessed. The parameters measured in spirometry were compared in each group before and after Ramadan and also between the two groups.

***Results:*** Spirometric measurements including forced expiratory volume in the first second (FEV1), forced vital capacity (FVC), peak expiratory flow (PEF), and FEV1/FVC were not significantly different before and after Ramadan in both groups of fasting and non-fasting patients. Furthermore, there was no significant difference between the two groups in terms of these spirometric parameters changes from baseline. Nevertheless, FEV1 change in the fasting group was significantly higher than that in the non-fasting group (1.46±5.37 vs. -0.13±3.08, respectively; *P*=0.040).

***Conclusion:*** The results of this study demonstrated that fasting has no significant effect on the severity of asthma and spirometric findings in patients with moderate to severe asthma. Therefore, fasting during Ramadan can be considered safe for patients with asthma.

## Introduction

 Asthma is a long-term condition affecting children and adults that is characterized by hyperresponsiveness, reversible airflow obstruction, and bronchial hyperresponsiveness.^1-3^ Based on the Iranian Asthma Association, the overall prevalence of asthma in Iran is estimated at 8-12% of the general population ^4,5^. A variety of strategies have been identified for the prevention of asthma attacks and reducing its symptoms and complications; however, the best strategy which is indicated by all established guidelines and treatment protocols is the regular use of medications along with a close follow-up, education of patients, and diminishing known risk factors.^6^

 Fasting is of paramount importance among many major religions worldwide, particularly Islam, and is a religious practice in which the faithful abstinence from eating and drinking from before sunrise (*dawn*) to sunset (*iftar*) should be continued for a month.^7-9^ Islamic fasting, like other Circadian rhythms, includes periods of “nutrition - abstinence from nutrition” which are repeated in a certain order, the way of repetition of these periods is such that the interval between the time of the sunset Adhan to the sunrise Adhan, which is the dark phase, is called the feeding phase, and the interval between the sunrise Adhan and the sunset Adhan, which is the phase of light, is called the phase of abstinence or abstinence from having food. In better words, Islamic fasting takes place in the form of rhythms and harmonic process.^10^

 Asthma is among the diseases whose symptoms’ beginning or exacerbation shows circadian variations in such a way that the exacerbation periods of asthma occur between midnight and early morning.^11^ Circadian changes in the lungs’ physiology and their volumes and inflammation of the airways can explain these changes in the symptoms and severity within the asthmatic patients.^12,13^

 Fasting during Ramadan can have a significant effect on the duration and outcomes of diseases by changing the pattern of medication consumption.^14^ According to the previous studies, 67% of Muslims, despite suffering from moderate to severe asthma, continue fasting in the holy month of Ramadan, and almost all patients use their medication before Sahar and after Iftar. On the other hand, the fasting duration change based on whether Ramadan is located in winter or summer, and as a consequence, the interval between Iftar and Sahar will be lower or langer than twelve hours.

 As far as we investigated, few studies have evaluated the influence of fasting on the exacerbation of asthma and variations in spirometric findings associated with conflicting outcomes.^14-16^ Finding a new circadian rhythm within the holy month of Ramadan and chronotherapy (coordination of biological rhythms with medical treatment) are the two main challenges that occur in Islamic fasting. In the current study, we aimed to evaluate the fasting effect in the holy month of Ramadan on spirometric parameters and respiratory symptoms in asthmatic patients.

## Materials and Methods

 The current study was conducted as a cohort study between May and June 2019 on 120 volunteer patients with moderate to severe asthma according to GINA guidelines referred to the lung sub-specialty clinics at Imam Reza Hospital in Tabriz. The sampling method was simple sampling and patients were included consecutively. The Sample size was calculated using G - Power software version 3.1.9.2 by considering an alpha error of 0.05 and study power of 0.8. Moreover, with an effect size equal to 0.5, an overall of 51 cases for each group resulted by calculation, which by considering the probability of loss to follow up, a final 60 individuals was considered adequate for each group (total of 120 cases).

 The inclusion criteria were as follows;

 Muslim patients aged in the range of 18-50, with a previous diagnosis of asthma by a physician, FEV1 less than 80%, presence of two or more asthma symptoms such as recurrent wheezing, cough, or chest tightness at rest, night, or early morning wheezing, activity-based cough or wheezing.

 The exclusion criteria were as follows;

 the presence of cardiovascular disease, hypertension, hypo/hyperthyroidism, pregnancy, lactating women, recent respiratory disease, abnormal chest graphic, chronic cardiovascular or known neuromuscular disease, malignancy, chest deformations, and abdominal or thoracic surgery.

 The included patients were categorized into two groups: 60 patients were fasting and 60 patients were non-fasting depending on whether they fast during the holy month of Ramadan. The study was performed according to the Declaration of Helsinki. Written informed consent was obtained from the participants. The demographic characteristics including age, gender, occupation, education, as well as the number of fasting days, symptoms, illness duration, and spirometric findings were also recorded in a pre-prepared checklist. The status of asthma control was assessed before and after Ramadan according to the Asthma Control Assessment Questionnaire. This questionnaire consisted of 5 items including asthma symptoms (nocturnal and daytime), the use of rescue medications, the effect of asthma on daily functioning, and the patient’s perception of asthma control over the previous 4 weeks with a score ranging from 1 to 5 for each item. Subsequently, responses for each of the 5 items were summed to yield a score ranging from 5 to 25. The scores above 20 were assumed as well-controlled, the score between 15-20 as poorly-controlled, and the score less than 15 as very poorly-controlled. Spirometry was conducted on all patients the day before and the day after Ramadan to assess pulmonary function. Before each spirometry, an explanation of the procedure was given to patients. In each patient, spirometry was performed three times to choose the most acceptable one.

 The statistical software of SPSS 24 was used to analyze the data. Paired t-test was implied to compare the quantitative data in each group, a chi-square statistical test was also utilized to compare qualitative data between the two groups, and an independent t-test was performed on the quantitative variables. *P* values below 0.05 were considered statistically significant.

## Results

 The average age of patients was 35.79 ± 9.08 years old. The average fasting duration was 26.13 ± 3.13 days in the fasting group. The minimum and maximum fasting durations were 20 and 30 days. The demographic and baseline data of the fasting and non-fasting groups are summarized in Table 1. The demographic characteristics and duration of the disease did not have a significant difference between the two groups. Moreover, there was no statistically significant difference in the spirometric parameters including forced expiratory volume in the first second (FEV1), forced vital capacity (FVC), peak expiratory flow (PEF), and FEV1/FVC in fasting before and after Ramadan (Table 2). Making a comparison between the obtained data from before and after Ramadan in non-fasting patients, no statistically significant difference was shown in terms of spirometric parameters including forced expiratory volume in the first second (FEV1), forced vital capacity (FVC), peak expiratory flow (PEF), and FEV1/FVC (Table 2). The changes in the results of the spirometric parameters from baseline (after Ramadan’s result – before Ramadan’s result) for both groups are described in Table 3. The changes in FVC, PEF, and FEV1/FVC did not have any significant difference between the two groups. Nevertheless, FEV1 change from baseline in the fasting group was significantly higher than that in the non-fasting group (1.46 ± 5.37 vs. -0.13 ± 3.08, respectively; *P* = 0.040).

**Table 1:** Demographic findings between fasting and non-fasting groups

		**Fasting**	**Non-fasting**	***P*** **-Value **
Age (year)		36.10 ± 9.47	35.48 ± 8.74	0.713
sex	male	40(%66.7)	28(%46.7)	0.135
	female	20(%33.3)	32(%53.3)	
Place of accomodation	city	59(%98.3)	60(%100)	0.312
	village	1(%1.7)	zero	
Educational level	illiterate	3(%5)	1(%1.7)	-----
	primary	3(%5)	1(%1.7)	
	Diploma	35(%58.3)	63(%63.3)	
	Bachelor	19(%31.7)	19(%31.7)	
	Masters and higher	zero	1(%1.7)	
Occupation	self-employed	30(%50)	31(%51.7)	-------
	Housewife	15(%25)	16(%26.7)	
	employee	9(%15)	10(%16.7)	
	mechanic	3(%5)	1(%1.7)	
	farmer	3(%5)	2(%3.4)	
Duration of asthma disease (years)		8.43 ± 1.57	7.10 ± 3.10	0.412

**Table 2:** Spirometric findings before and after Ramadan between fasting and non-fasting patients

	**Fasting**			**Non-fasting**		
	**before Ramadan**	**after Ramadan**	***P*** ** Value**	**before Ramadan**	**after Ramadan**	***P*** ** Value**
FEV1	83.19 ± 15.46	83.85 ± 18.27	0.140	83.65 ± 18.01	83.36 ± 17.37	0.350
FVC	90.14 ± 16.27	90.48 ± 14.58	0.751	88.17 ± 13.39	86.99 ± 16.63	0.381
PEF	67.14 ± 27.96	66.92 ± 24.56	0.901	69.54 ± 28.05	68.67 ± 26.23	0.160
FEV1/FVC	81.11 ± 4.63	80.06 ± 10.69	0.440	81.70 ± 5.66	81.57 ± 5.91	0.510

**Table 3:** The changes in spirometric findings after Ramadan compared to before Ramadan in fasting and non-fasting asthmatic patients

	**Fasting**	**Non-fasting**	***P*** ** Value **
FEV1 change (%)	1.5 ± 46.37	-0.13 ± 3.08	0.040
FVC change (%)	1.46 ± 11.65	-1.24 ± 11.87	0.201
PEF change (%)	3.42 ± 22.53	-0.59 ± 3.80	0.174
FEV1/FVC change (%)	-1.17 ± 12.62	-0.16 ± 2.18	0.543

**Figure 1:**
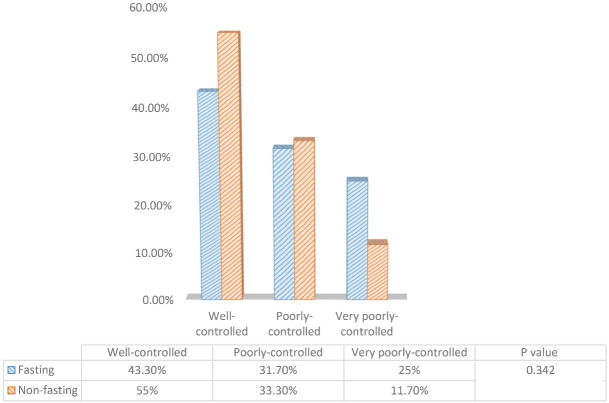
Asthma control diagram according to asthma control questionnaire before Ramadan between two fasting and non-fasting groups.

**Figure 2:**
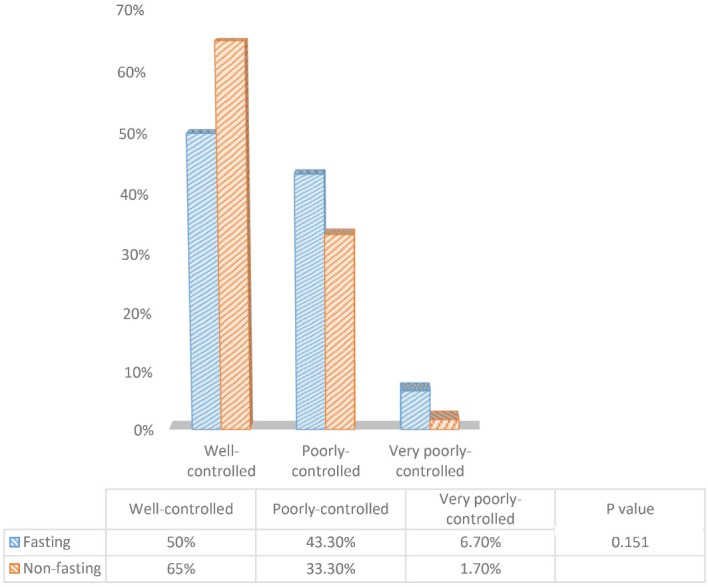
Asthma control diagram according to asthma control questionnaire after Ramadan between two fasting and non-fasting groups.

 The results of the asthma control questionnaire before and after Ramadan between the fasting and non-fasting group are depicted in Figures 1 and 2, respectively. There was no significant difference in terms of asthma control status before Ramadan between fasting and non-fasting groups. Despite the increased cases of well-controlled asthma in the non-fasting group after Ramadan (55% to 65%), this difference was not statistically significant (*P* = 0.151).

## Discussion

 One of the oldest acts of worship in Islam is fasting during the holy month of Ramadan, which is common among the ethnicities and nations of the world and is a divine duty for all Muslims.^17^

 During the period from Sahar to Iftar, Muslims are forbidden from eating and drinking during this month. Besides in this month, the sleep-wake cycle is disrupted and hence it may have some effects on the body systems.^17^

 Previous researches have reported the positive and negative influences of fasting on some diseases such as diabetes and other endocrine diseases.^18^ On the other side, other studies have reported fasting may worsen or improve symptoms in asthmatic patients. In this study, we evaluated the effect of fasting within the holy month of Ramadan in asthmatic patients using spirometry and the Asthma Control Assessment Questionnaire. Spirometric results did not indicate a statistically significant difference among fasting and non-fasting groups before and after Ramadan. Nevertheless, FEV1 had more change from baseline (before Ramadan) and larger improvement in fasting patients compared to the non-fasting ones, and this may show a potential positive effect of fasting in asthma improvements, although further randomized clinical trials are warranted to support this idea. The observed positive effect of fasting on FEV1 can be a result of weight loss during Ramadan as the higher level of cytokines and inflammatory mediators such as Interleukin-6, TNF-alpha, eotaxin, leptin in obese contribute to the development or increased clinical expression of asthma in promoting airway inflammation. Therefore, weight loss during Ramadan may improve FEV1 in asthmatic patients. Weight changes during Ramadan have been identified as one of the reasons for changes in respiratory markers during this month ^19^. In a study by Hakala et al on 15 overweight asthmatic patients, a low-calorie diet led to weight loss resulting in a decrease in the amount of airway constriction.^20^ Furthermore, due to the lower level of energy in fasting patients, they are inclined to stay more in indoor environments and rest until iftar. Therefore, they will have less contact with allergens during Ramadan. Consequently, a lower inflammatory response due to lower contact with allergens can be considered as another mechanism for improved FEV1 during Ramadan. Moreover, some other mechanisms that are recognized in some studies to be able to modify lung function and alleviate disease severity during the holy month of Ramadan can be considered for justifying this positive effect, including the reduction of food allergens, reduction of physical activity, low stomach volume which impose less pressure on the diaphragm, smoking cessation, and reduction of gastroesophageal reflux disease. Additionally starvation causes physical stress which leads to further release of catecholamines and consequently the expansion of airways.^16^

 Similar results have been reported by Subhan et al ^21^ with the increase in the FEV1 after Ramadan. Nevertheless, Mousavi et al showed an increase in the lung capacity of healthy people during Ramadan which is a result of reduced lung recoil, increased outward recoil of the chest wall, and increased strength of contraction of the inspiratory muscles.^22^ On the other hand, Norozi et al have demonstrated that among the lung function tests, peak expiratory flow (PEF) improved within 29 patients with controlled asthma after Ramadan (PEF decreased from 13% to 10% after 4 weeks of fasting).^15^ In a study on 1590 asthmatic patients for 4 years, Bener et al did not observe any significant change in all pulmonary function tests during Ramadan compared to the previous or subsequent months.^23^ No difference between the results of pulmonary tests and the severity of asthma was observed in the study of Adeli et al before and after Ramadan among asthma patients.^16^ Amini et al reported similar results in a study on the asthmatic patients, except for peak expiratory flow rate (PEFR), which increased during the fasting period.^15^ Askari et al also found that fasting had positive effects during Ramadan on asthma.^6^ Several hypotheses have been indicated to justify the variations in the findings of studies, including cultural and economic differences among the countries, hormonal variations between men and women, season changes in the month of Ramadan, different diets, and general health status among the evaluated patients in these studies.^24,25^

 Altogether, considering that asthma control status remained relatively unchanged during Ramadan, our observations support the idea that fasting can be considered safe for asthmatic patients. Some studies have also reported no significant changes in asthmatic patients’ symptoms such as dyspnea, wheezing, cough, and chest tightness by fasting.^6,26^ Erkekol et al indicated that asthma is not a barrier for asthmatic patients against fasting during Ramadan, and these patients, by continuing their medications and treatments habits can safely fast during the holy month of Ramadan.^26,27^

 It has been similarly expressed in other lung diseases that fasting does not have any impact on spirometry findings. Zouraei et al in their study on male COPD patients indicated that fasting during Ramadan does not have any impact on spirometry parameters.^28^ Some other studies have examined the fasting effect on healthy non-asthmatic individuals and reported no significant effect by fasting in these participants. Siddiqui et al observed no changes in the respiratory test data of 46 healthy men during Ramadan. Nevertheless, due to BMI increase and weight regain in patients after a short period after Ramadan (by returning to a regular diet) FVC had decreased in these patients.^29^

## Conclusion

 The results of this study demonstrated that fasting has no significant effect on the severity of asthma and spirometric findings in patients with moderate to severe asthma. Therefore, fasting during Ramadan can be considered safe for patients with asthma.

## Competing interests

 None.

## Ethical approval

 The protocol of this study was approved by the ethics committee of the Tabriz University of Medical Sciences with the ethics code IR.TBZMED.REC.1398.485.

## Funding

 This study was supported by a fund from the Tabriz University of Medical Sciences.
